# Arf4 Is Required for Mammalian Development but Dispensable for Ciliary Assembly

**DOI:** 10.1371/journal.pgen.1004170

**Published:** 2014-02-20

**Authors:** John A. Follit, Jovenal T. San Agustin, Julie A. Jonassen, Tingting Huang, Jaime A. Rivera-Perez, Kimberly D. Tremblay, Gregory J. Pazour

**Affiliations:** 1 Program in Molecular Medicine, University of Massachusetts Medical School, Biotech II, Worcester, Massachusetts, United States of America; 2 Department of Microbiology and Physiological Systems, University of Massachusetts Medical School, Worcester, Massachusetts, United States of America; 3 Department of Cell and Developmental Biology, University of Massachusetts Medical School, Worcester, Massachusetts, United States of America; 4 Department of Veterinary and Animal Sciences, University of Massachusetts, Amherst, Amherst, Massachusetts, United States of America; Washington University School of Medicine, United States of America

## Abstract

The primary cilium is a sensory organelle, defects in which cause a wide range of human diseases including retinal degeneration, polycystic kidney disease and birth defects. The sensory functions of cilia require specific receptors to be targeted to the ciliary subdomain of the plasma membrane. Arf4 has been proposed to sort cargo destined for the cilium at the Golgi complex and deemed a key regulator of ciliary protein trafficking. In this work, we show that Arf4 binds to the ciliary targeting sequence (CTS) of fibrocystin. Knockdown of Arf4 indicates that it is not absolutely required for trafficking of the fibrocystin CTS to cilia as steady-state CTS levels are unaffected. However, we did observe a delay in delivery of newly synthesized CTS from the Golgi complex to the cilium when Arf4 was reduced. *Arf4* mutant mice are embryonic lethal and die at mid-gestation shortly after node formation. Nodal cilia appeared normal and functioned properly to break left-right symmetry in *Arf4* mutant embryos. At this stage of development Arf4 expression is highest in the visceral endoderm but we did not detect cilia on these cells. In the visceral endoderm, the lack of Arf4 caused defects in cell structure and apical protein localization. This work suggests that while Arf4 is not required for ciliary assembly, it is important for the efficient transport of fibrocystin to cilia, and also plays critical roles in non-ciliary processes.

## Author Summary

Primary cilia are ubiquitous sensory organelles that play vital roles in an ever-growing class of human diseases termed ciliopathies including obesity, retinal degeneration and polycystic kidney disease. The proper function of the primary cilium relies on a cell's ability to target and concentrate specific receptors to the ciliary membrane – a unique subdomain of the plasma membrane yet little is known about how receptors are trafficked to the primary cilium. Mutations affecting the ciliary localized receptor fibrocystin (*PKHD1*) cause autosomal recessive polycystic kidney disease, which affects approximately 1∶20,000 individuals. Previously we identified a motif located in the cytoplasmic domain of fibrocystin that is required for its ciliary localization. In this work we demonstrate that the ciliary targeting sequence (CTS) of fibrocystin interacts with the small G protein Arf4 and this interaction is important for the efficient delivery of the CTS to cilia in cultured cells. Disruption of *Arf4* in mice results in defects in the non-ciliated visceral endoderm and death at mid-gestation indicating Arf4 has vital functions in addition to ciliary protein trafficking.

## Introduction

Cilia play diverse motility and sensory functions throughout the eukaryotic kingdom, but play especially critical roles in vertebrates where severe defects lead to embryonic lethality while mild defects cause a wide range of syndromes affecting every organ system. Both the motility and sensory functions of cilia are important for health and development, but it is now recognized that sensory defects underlie the most severe maladies affecting humans. The sensory functions of cilia rely on a cell's ability to target and concentrate a specific set of receptors to the ciliary membrane. While contiguous with the plasma membrane of the cell, the ciliary membrane is a distinct compartment to which the cell targets and concentrates a unique complement of proteins [1], [2]. The list of membrane proteins found in the ciliary compartment is constantly growing; among the most studied ciliary proteins are the polycystins and fibrocystin that are defective in human polycystic kidney disease, rhodopsins and opsins that are critical for vision and the patched and smoothened receptors of the hedgehog pathway.

The mechanism that targets membrane proteins specifically to the ciliary compartment is an active area of study but very little is definitively known [3]. It appears that ciliary membrane proteins contain cis-acting motifs that cause them to be localized to cilia. We identified one of these ciliary targeting sequences (CTS) in fibrocystin, the gene product of the human autosomal recessive polycystic kidney disease gene (*PKHD1*) [4]–[7]. Like many other CTSs, the fibrocystin CTS contains lipid-modified residues that target the protein to lipid rafts, which appears to be part of the ciliary trafficking pathway. We proposed that this sequence might interact with proteins that are important for sorting or transport to the ciliary membrane compartment. In support of this idea, we found that the fibrocystin CTS interacted with Rab8, a protein widely recognized as important to ciliary membrane protein trafficking [7]–[9]. In the present work we asked if the fibrocystin CTS could interact with Arf4 as work of Deretic and colleagues has shown that this protein interacts with the CTS of opsin and is important for the formation of rhodopsin carrier vesicles at the Golgi complex [10], [11].

Arf4 is a small G protein in the Arf subfamily of Ras-related small G proteins. Mice have six members of this family while humans have lost Arf2 and have five members. Arf1 and Arf6 have been best-studied and are thought to organize membrane protein cargos into coated vesicles for transport to specific lipid domains in the cell [12]–[15]. Arf1 forms coated vesicles at the Golgi complex crucial for trafficking between the ER and Golgi and throughout the cell while Arf6 is thought to operate at the plasma membrane and regulate endosomal-membrane traffic. Arf4, which was thought to evolve from an Arf1-like precursor when metazoans arose, is a relatively unstudied member of the family [13]–[15]. Arf4 was first proposed to be important for ciliary trafficking when it was found to interact with the C-terminal tail of rhodopsin where the CTS is localized [10]. Depletion of Arf4 from an *in vitro* budding assay showed that it was important for the formation of rhodopsin carrier vesicles [11]. The CTSs of rhodopsins are contained in the last five amino acids (QV[S/A]PA) at the C-terminal end of the protein. The V and P residues are mutated in human patients with autosomal dominant retinitis pigmentosa. These residues were found to be important in an *in vitro* assay for the formation of rhodopsin carrier vesicles, thus the sequence has become known as a VXPX motif. A similar RVXP motif is present in the CNGB1b subunit of the CNG channel, another ciliary-localized protein [16]. The VXPX sequence is part of the CTS in polycystin-1 and polycystin-2 and it is hypothesized that VXPX motifs function as Arf4 binding sites for transport to the cilium [12], [17], [18].

In this work we ask if Arf4 plays a role in trafficking the fibrocystin CTS to the cilium and probe the function of Arf4 *in vivo* by analyzing a mutant mouse. Although the fibrocystin CTS does not contain a VXPX motif, it does bind to Arf4. Arf4 is not required for the trafficking of the fibrocystin CTS to cilia, but knockdown of Arf4 increases the time needed for the protein to travel from the Golgi complex to the cilium. Deletion of *Arf4* in the mouse does not affect the formation or function of nodal cilia, but causes embryonic lethality at mid-gestation, probably due to trafficking defects in the visceral endoderm.

## Results

### Arf4 Interacts with the Ciliary Targeting Sequence of Fibrocystin

**Figure 1:**
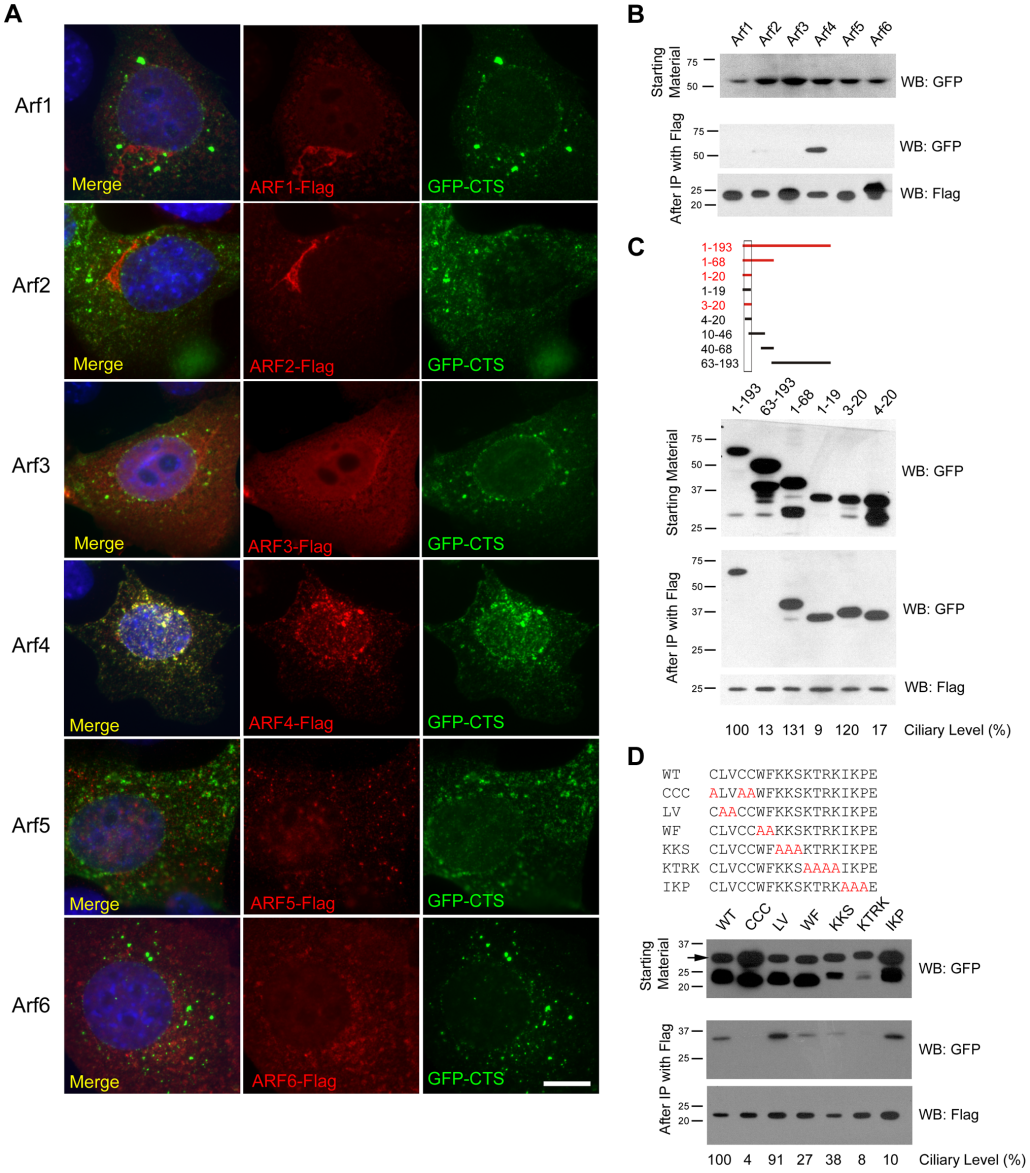
Arf4 interacts with the ciliary targeting sequence of fibrocystin. A. Mouse IMCD3 cells co-transfected with FLAG-tagged Arf proteins and GFP-tagged CTS; DAPI (blue) anti-FLAG (red) and GFP (green). Scale bar is 10 µm. Note extensive colocalization of Arf4 and CTS-GFP. B. FLAG IPs from cells shown in A were analyzed by western blot after SDS-PAGE. The top panel shows GFP-CTS expressed in the starting material. The bottom panel indicates that each of the 6 Arf proteins was precipitated. Only Arf4 brought down significant amounts of GFP-CTS (middle panel). C. Selected CTS deletion constructs [7] were co-expressed with Arf4-FLAG. Following FLAG IP, Arf4 (bottom panel) precipitated the full-length intracellular tail of fibrocystin (1–193) and also precipitated truncations including the CTS but not the 63–193 construct, which lacks the CTS. Deletions to the CTS that prevent ciliary trafficking (1–19) and (4–20) also bound Arf4. Ciliary Level is a relative measure of the ability of the fusion protein to traffic to cilia with the control construct set to 100%. Data are from [7]. D. Alanine scanning mutant CTSs [7] were co-expressed with Arf4-FLAG. After Arf4 precipitation (bottom panel), WT, LV and IKP mutant CTSs were brought down (middle panel). Mutations that completely prevented ciliary targeting of the CTS (CCC and KTRK) failed to interact with Arf4. Ciliary Level is as described in C.

Fibrocystin (polyductin), the human autosomal recessive polycystic kidney disease gene product, is targeted to cilia by an 18-residue ciliary targeting sequence (CTS) located in the cytoplasmic C-terminal tail of the protein. Previously we showed that this sequence interacted with Rab8 and proposed that it may function by interacting with proteins involved in the sorting and transport of ciliary membrane proteins [7]. Recent work indicates that Arf4 is required for trafficking of other ciliary proteins including rhodopsin and the polycystins [10], [11], [18]. Arf4 is one of six members of the Arf subfamily of small G proteins. Our previous analysis showed that in non-ciliated cells, the GFP-CTS localized to small puncta that appeared to be lipid microdomains rich in GM1 gangliosides. Interestingly, Arf4 exhibited significant co-localization with the CTS-GFP in these puncta while the rest of the Arf family did not (Figure 1A). To determine if this colocalization represented a physical interaction, we immunoprecipitated each of the Arfs and asked if the CTS-GFP was co precipitated. Arf4 strongly precipitated the CTS, while the other Arfs either precipitated no CTS (Arf1, 3, 5, 6) or only a small amount (Arf2) (Figure 1B). Because *ARF2* is a pseudogene in humans we did not pursue the Arf2 interaction any further. The failure of the CTS to interact with Arf5 suggests that the Arf4 interaction is likely to be specific as Arf4 and Arf5 share over 90% identity at the amino acid level.

To further characterize the interaction between Arf4 and the CTS we tested the ability of a series of CTS deletion constructs to interact with Arf4 in the co-expression immunoprecipitation assay (Figure 1C). In this assay, the CTS is sufficient for binding as the 3–20 construct was precipitated by Arf4 while the 63–193 construct, which lacks the CTS, was not bound. The 1–19 and 4–20 constructs also interact with Arf4 but do not target to cilia, indicating the CTS has functions in addition to Arf4 binding.

Next we examined the ability of Arf4 to interact with a series of alanine-scanning mutations within the CTS (Figure 1D). In general, the ability of Arf4 to bind the CTS correlated with the ability of the CTS to traffic to cilia. For example, mutations affecting the palmitoylated cysteines (CCC>AAA) or conserved basic residues (KTRK>AAAA) prevent ciliary targeting and likewise inhibit Arf4 binding. Mutations that exhibit little effect on the ciliary targeting of the CTS (LV>AA) similarly do not affect Arf4 binding. However the concordance was not perfect as the (IKP>AAA) mutation blocked ciliary targeting but had little effect on Arf4 binding, again supporting the idea that while the CTS is an Arf4 binding site, this is not its entire function.

Arf4 cycles between GTP and GDP bound states. To explore the role of this cycling on the ciliary trafficking of the CTS we measured the effects of constitutively active and dominant negative Arf4 on ciliary targeting and interaction with the CTS (Figure S1). Constitutively active Arf4 (Q67L) co-localized with IFT20 at the Golgi Complex while wild type or dominant negative (T13N, T48N) Arf4 displayed a punctate distribution in the cytoplasm (Figure S1A). The mutant forms of Arf4 retained the ability to bind the CTS (Figure S1E). Expression of dominant negative Arf4 reduced the percent of ciliated cells (Figure S1B) and ciliary length (Figure S1C). Surprisingly, over-expression of any Arf4 constructs completely inhibited CTS trafficking to the cilium (Figure S1D). These data indicate that increases in Arf4 levels prevent ciliary trafficking of the CTS possibly by sequestering it in the cytoplasm.

### Arf4 Is Required for Efficient Delivery of the Fibrocystin C Terminal Tail to the Cilium

**Figure 2:**
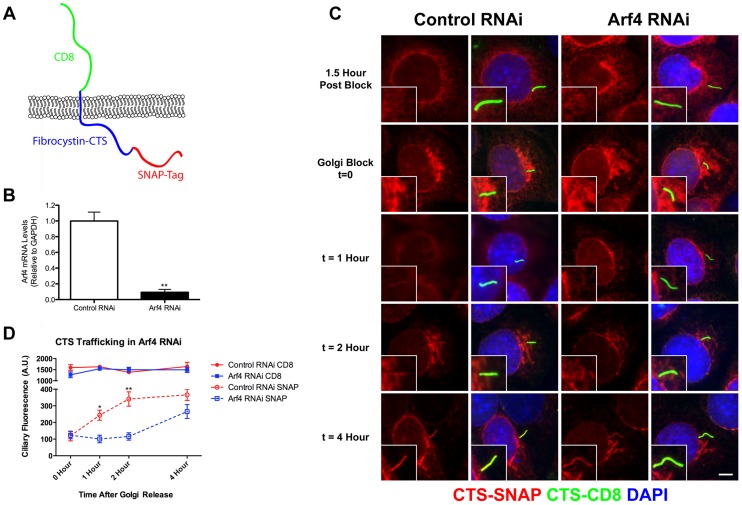
Arf4 knockdown delays CTS trafficking to the primary cilium. A. Diagram of the CD8-CTS-SNAP construct used in the trafficking assays. The extracellular domain of CD8 (green) is fused to fibrocystin (blue) just N-terminal to its transmembrane domain and the SNAP-tag (red) is fused to the C-terminus of fibrocystin. B. siRNA mediated knockdown of Arf4 results in a >90% reduction in Arf4 mRNA abundance as indicated by qRT-PCR. C. Immunofluorescence images showing CTS-SNAP trafficking in control (left two columns) and Arf4 knockdown (right two columns) cells. Mouse kidney cells stably expressing CD8-CTS-SNAP were blocked with non-fluorescent SNAP substrate, allowed 1.5 hr to synthesize new CD8-CTS-SNAP (1.5 Hour Post Block, top row), treated with cycloheximide and incubated at 19°C for 2 hr to accumulate CD8-CTS-SNAP at the Golgi complex (Golgi Block t = 0, second row). At t = 0 cells were returned to 37°C for the indicated amount of time (bottom 3 rows). At indicated times cells were fixed and stained with DAPI (blue) anti-CD8 (green) and TMR-SNAP (red). Scale bar is 5 µm. Insets are 2X enlargements of the cilium. D. Quantification of the delivery of CD8-CTS-SNAP to cilia. Starting at the time of temperature shift from 19°C to 37°C (t = 0), the ciliary levels of CD8 and SNAP were measured by fluorescence microscopy. CD8 staining (green) is present at similar levels in the control and knockdown cells at all time points. Newly synthesized CD8-CTS-SNAP (red) is seen in control cilia within 1 hr of release. An increase is observed at 2 hr and the amount of SNAP label plateaus at 4 hr. Knockdown of Arf4 delays delivery such that little label is detected in the cilia until the 4 hr time point. Differences were significant at t = 1, 2 (t = 0 NS; t = 1 * p<0.05; t = 2 **p<0.005; t = 4 NS). The mean CD8 fluorescence (solid line) and SNAP fluorescence (dotted line) were plotted from three independent experiments in which 50 cilia were quantitated for each condition (Arf4 knockdown and control) at each time point. Error bars represent standard error of the mean.

Prior studies in frog photoreceptors [10], [11] and cultured mammalian cells [18] suggest that Arf4 functions at the Golgi complex to direct rhodopsin and polycystin-1 to the cilium. Our initial immunofluorescence and biochemical results suggested that Arf4 may also be required for the ciliary targeting of fibrocystin. To test the role of Arf4 in the trafficking of fibrocystin, we developed a pulse chase assay to measure its movement through the endomembrane system and delivery to the cilium. To do this, we created a chimeric molecule containing the extracellular domain of CD8 fused to the C-terminal tail of fibrocystin (including the transmembrane domain) with a SNAP tag [19] on the C-Terminal end (Figure 2A) and expressed this in mouse kidney collecting duct cells (IMCD3). The extracellular domain of CD8 contains a signal sequence that when combined with the transmembrane domain of fibrocystin produces a type 1 membrane protein with membrane topology the same as native fibrocystin except that the large (3,851 residue) extracellular domain is replaced with the CD8 epitope. The SNAP tag is a fragment of the DNA repair protein O^6^-alkylguanine-DNA alkyltransferase that can be covalently modified with benzyl guanine derivatives and allows for pulse chase experiments [20], [21]. We developed a protocol to follow the movement of this chimeric protein through the endo-membrane system. At the beginning of the experiment all existing SNAP sites are blocked with a non-fluorescent benzyl-guanine so that only newly synthesized protein will be labeled by the fluorescent benzyl-guanine. The newly synthesized SNAP-CTS is first detected in the endoplasmic reticulum as expected for a trans-membrane receptor. The protein then moves to the Golgi complex where it can be trapped using a 19°C temperature block [22]. Shifting the cells back to 37°C allows the accumulated protein to exit the Golgi and traffic to the cilium where it can be detected within 30 min of release.

To determine if Arf4 is involved in trafficking of the CTS, cells expressing the CD8-CTS-SNAP construct were treated with siRNA to reduce the level of Arf4. Arf4 mRNA level was reduced greater than 90% as compared to cells treated with a control scrambled siRNA (Figure 2B). The Arf4 knockdown did not affect the percent of ciliated cells nor did it affect ciliary length (data not shown). Arf4 knockdown did not affect the total amount of CD8-CTS-SNAP in the cilium as measured by CD8 fluorescence (Figure 2D). To determine if the reduction of Arf4 affected the rate of delivery to the cilium, we measured the time that it takes for the CD8-CTS-SNAP construct to move from the Golgi complex to the cilium by using the pulse chase protocol described above. Newly synthesized protein was accumulated in the Golgi complex by a 19°C block and then released by shifting cells to 37°C (Figure 2 C, D). In control cells, after release from the Golgi block the CD8-SNAP-CTS moves quickly to the cilium and is detectable at the 1 hr time point with the ciliary level peaking at about 2 hrs post block (Figure 2C, insets). In contrast, when Arf4 is depleted, little CD8-CTS-SNAP is detectable in the cilium within the first two hrs after release from Golgi block but protein is detectable at 4 hrs. These data indicate Arf4 is not absolutely required for the delivery of the CTS to the cilium but does play a kinetic role in the steps between the Golgi complex and the cilium.

### Arf4 Mutant Mice Are Embryonic Lethal

**Figure 3:**
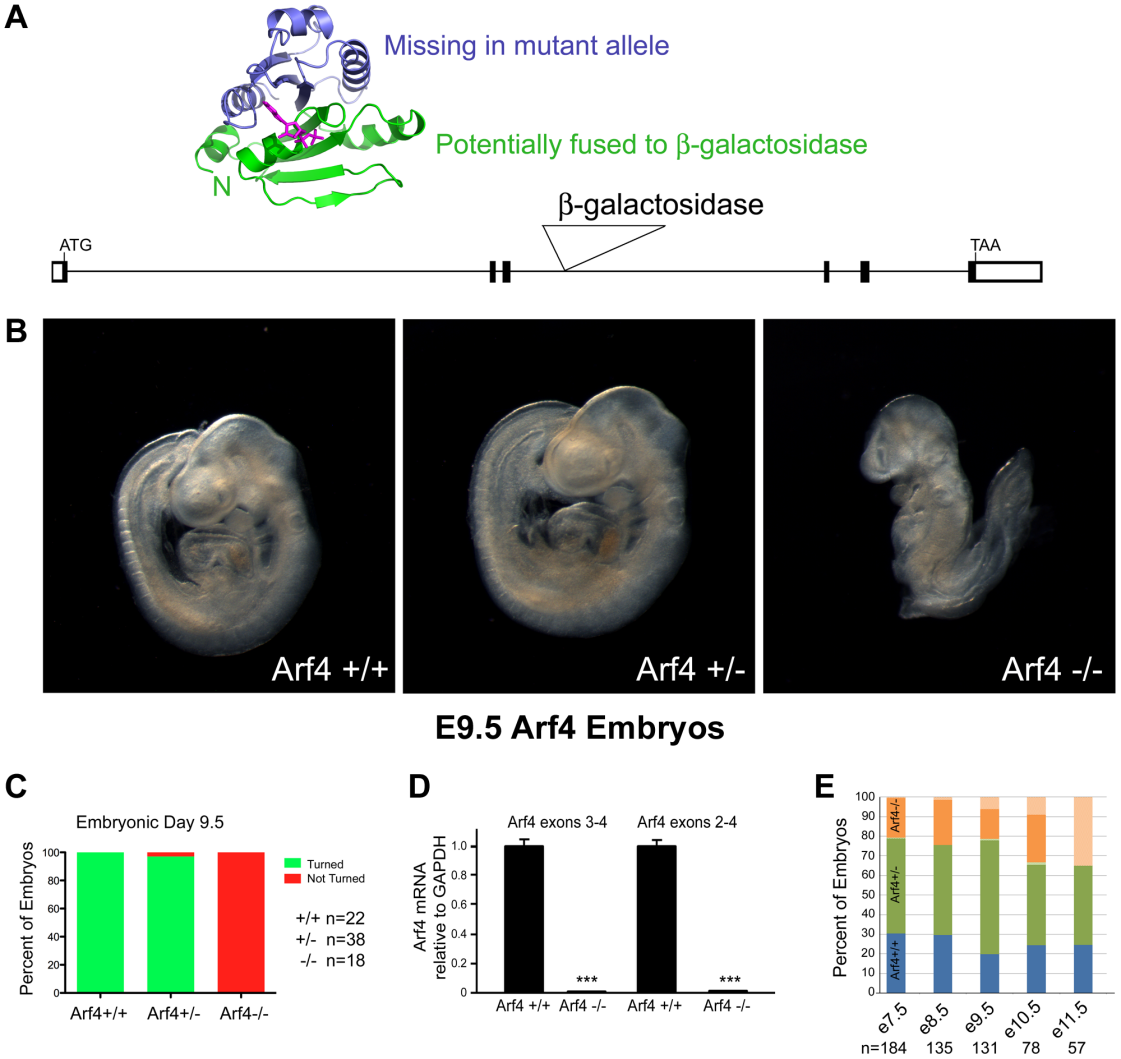
Arf4 mutant mice are embryonic lethal. A. Schematic of the gene trap allele analyzed and the potential protein. The *Arf4* allele contains a β-galactosidase insertion in exon 3. 89 residues (green in the ribbon diagram) are potentially translated from this allele and may be fused to the N-terminus of β-galactosidase. B, C. By E9.5, wild type and most heterozygous mice complete embryonic turning and adopt the normal fetal orientation. At this time point, Arf4 mutants are smaller and most have not completed embryonic turning. D. qRT-PCR analysis using primers that span the insertion site in intron 3 indicates that mean Arf4 mRNA abundance in the mutant embryos is strongly reduced; error bars depict standard error of the mean. ***p<0.001. E. Genotype distribution as a function of age (+/+ blue; +/− green; −/− orange; dead embryos are depicted by a lighter shade). Arf4 mutant embryos are present until E11.5, however all mutant embryos dissected after E10.5 were dead. Number of embryos (n) analyzed at each time point is given below the graph.

Our initial data indicates that Arf4 interacts with the CTS of fibrocystin and this interaction is required for the efficient delivery of newly synthesized CTS to the cilium. Data in the literature suggests thatArf4 is important for the trafficking of rhodopsin and the polycystins to cilium and it has been suggested that it is a global regulator of ciliary cargo [10], [11], [18]. To determine if Arf4 is a global regulator of ciliary protein trafficking we created an *Arf4* genetrap mouse with the prediction that if it plays this role, the mouse should have ciliopathy phenotypes. Embryonic stem cells harboring a LacZ insertion in the *Arf4* locus just downstream of exon 3 were obtained from the Sanger Institute and used to create an *Arf4* genetrap mouse (Figure 3). The allele we generated expresses less than 1% of control *Arf4* mRNA and is, at minimum, a strong hypomorph (Figure 3D).

Mice lacking cilia typically die mid-gestation around day embryonic day (E) 10 with a failure to undergo embryonic turning and have severe disruptions in left-right patterning [23]. Similar to this, the *Arf4* mutant mice are embryonic lethal at mid-gestation with no live embryos detected after day E10.5 (Figure 3E). At E9.5 the mutant embryos were smaller than either wild type or heterozygous embryos and almost never completed embryonic turning to assume the characteristic fetal body position that is observed in almost all wild type and heterozygote embryos by this time (Figure 3B, C).

**Figure 4:**
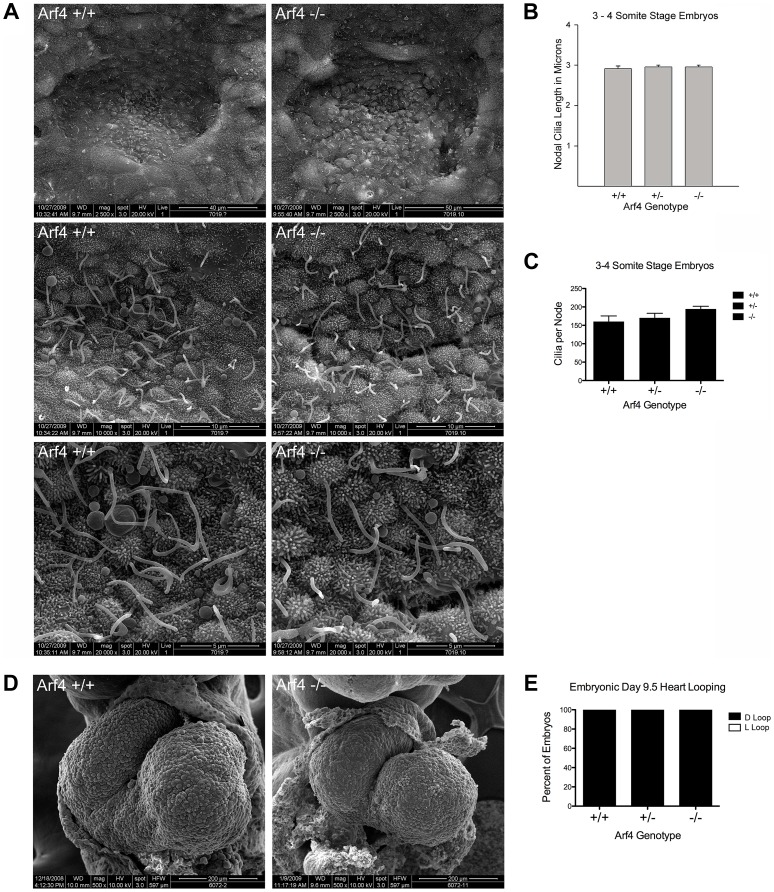
Arf4 mutant mice have functional nodal cilia. A. SEM of 3–4 somite developmentally matched embryos at 2,500× (top), 10,000× (middle) and 20,000× (bottom) magnification showing nodal cilia in wild type (left column) and Arf4 mutant (right column) embryos. B. Nodal cilia length is not significantly affected by the Arf4 mutation. Mean cilia length from 3–4 somite stage embryos is plotted (N = 3 each genotype); error bars are standard error of the mean. C. Total number of nodal cilia is not significantly affected in the Arf4 mutants. Mean number of cilia per node (N = 3 for each genotype) is plotted; error bars are standard error of the mean. D. SEM of developing heart tube at E9.5 showing normal heart looping in wild type (left) and Arf4 mutant (right) embryos. E. Heart looping analysis of E9.5 embryos shows that all had normally looped hearts (n = 22, 38, 18 for +/+, +/−, −/−).

To investigate if Arf4 affects ciliary assembly we performed scanning electron microscopy (SEM) of the node, which is thought to be the first ciliated structure in the embryo. To preclude differences caused by a developmental delay in the mutant embryos, embryos from multiple litters were dissected and those at the 3–4 somite stages were examined by SEM (Figure 4A). No differences were observed in either length or number of cilia present on the mutant nodes as compared to wild type or heterozygous embryos (Figure 4B, C) indicating Arf4 is not required for ciliary assembly. Nodal cilia beat to create a leftward flow required to break the left/right symmetry of the developing embryo [23]. This leads to asymmetric gene expression patterns on the left versus right side of the embryos and eventually leads to the right-right pattern of the abdominal and visceral organs. One of the earliest physical manifestations is the looping of the heart tube, which under normal conditions adopts a characteristic D-loop by day E9.5. The developing heart in *Arf4* mutant embryos always adopts a D-loop indicating the nodal cilia present in the *Arf4* mutant embryos are functional in breaking left/right symmetry (Figure 4D, E).

### Arf4 Expression Is Highest in the Visceral Endoderm

**Figure 5:**
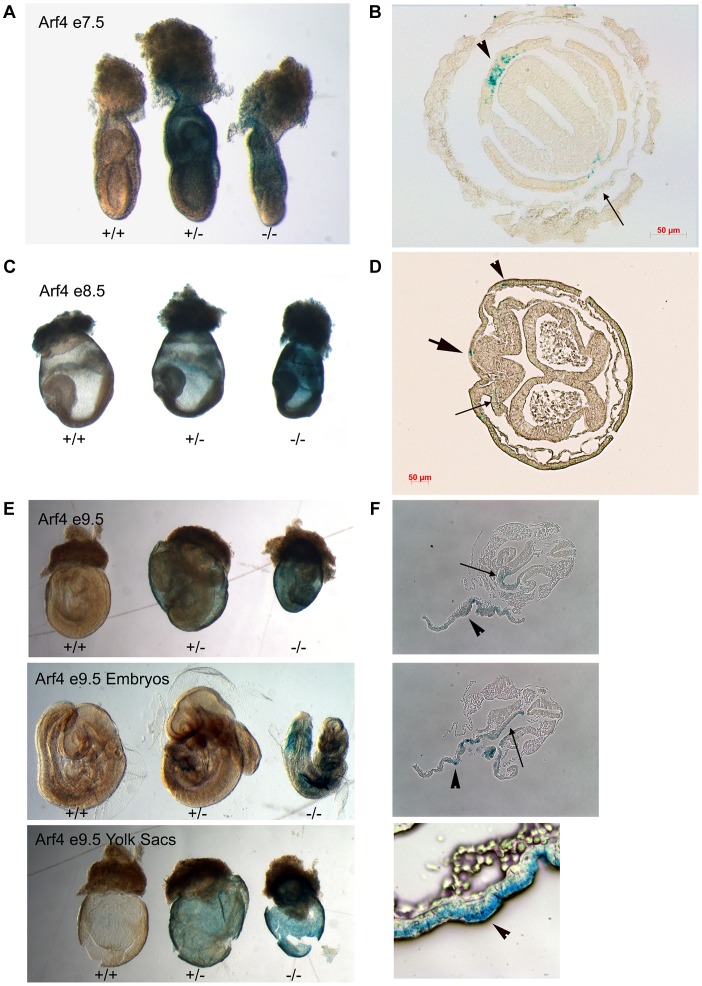
Arf4 expression is concentrated in the visceral endoderm during development. Whole mount β-Galactosidase stained embryos (A, C, E) and sections of stained mutant embryos (B, D, F). On E7 (A, B), Arf4-β-Gal expression is highest in the visceral endoderm (arrow head) and is seen at lower levels in the parietal endoderm (small arrow, see Figure S2 for more planes). On E8 (C, D) expression remains in the visceral endoderm (arrow head) and is seen in the forming definitive endoderm (large arrow) and the paraxial mesoderm (small arrow, see Figure S3 for more planes). On E9 (E, F), the strongest expression is seen in the visceral endoderm layer of the yolk sac (F, arrow heads) but staining is also observed in the embryo. In the embryo, the staining is strongest in the endoderm of the liver bud (F. top panel, small arrow) and the developing gut tube (F, middle panel, small arrow).

*Arf4* mutant mice die between embryonic days 9 and 10. This embryonic lethality does not appear to be connected to ciliary dysfunction leaving the cause of death unknown. To identify the site of pathology, we took advantage of the β-galactosidase insertion that was used to generate this allele and performed X-Gal staining to identify the sites of high *Arf4* expression (Figure 5). As expected, no staining was observed in the wild type embryos. In the mutants and heterozygotes, the majority of the staining was observed in the visceral yolk sac with only mutants exhibiting label in the embryo on E8 and E9 (Figure 5). At day 8, the label was observed in the allantois, paraxial mesoderm and in the forming definitive endoderm in hindgut region (Figure 5C, D; Figure S2). By E9.5 the definitive endoderm in the posterior foregut, including the liver bud was most strongly stained (Figure 5F). The yolk sac consists of two layers; the outer visceral endoderm is composed of highly polarized cells covered with microvilli on their apical surface, while the inner mesoderm gives rise to the developing blood islands in early development of the circulatory system [24]. The β-galactosidase activity was exclusively found in the visceral endoderm layer of the yolk sac (Figure 5F, bottom row).

### Arf4 Is Required for Visceral Endoderm Function

**Figure 6:**
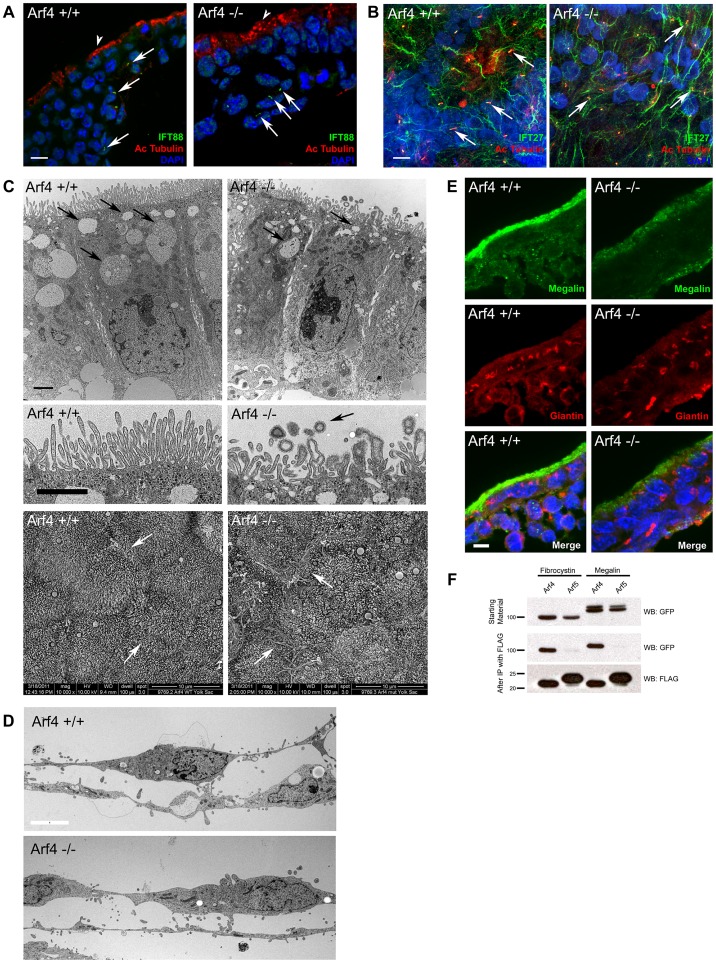
Arf4 mutant embryos have defects in the visceral endoderm. A. Confocal images of the visceral endoderm (arrowhead, outermost cell layer) and adjacent mesoderm (inner cell layers) of E8.5 embryo sections. Cilia (arrows) stained with acetylated tubulin (red) and IFT88 (green) are present on mesodermal cells in both mutant and wild type embryos. No cilia were observed on the visceral endoderm. Scale bar is 5 µM. B. Confocal images of flat mounted yolk sacs from E9.5 embryos stained with acetylated tubulin (red) and IFT27 (green). Numerous cilia (arrows) are present on the mesoderm in both wild type and mutant embryos. Scale bar is 5 µM. C. Thin section TEM images of the visceral endoderm at E8.5 of wild type (left) and Arf4 mutant (right) embryos. Healthy visceral endoderm is filled with large apical vacuoles or lysosomes (arrows, top panel), which in the mutants are reduced in number and size. The microvilli on the apical surface of the visceral endoderm (middle panel) are abnormally organized and shaped in the mutant, and are associated with small coated vesicles (arrow) not seen in the controls. Scale bar is 2 µM for all TEM images. The abnormally shaped microvilli are better observed by SEM (bottom panels). In controls, the microvilli have a uniform diameter while the tips are bulbous in the mutants. In addition, cell/cell contacts appear to be compromised in the mutants, as the microprojections that normally form the interdigitations between the lateral surfaces of the cells are visible on the apical surface (regions of cell/cell contact are marked with arrows). D. Thin section TEM images of the amnion at E8.5 of wild type (top) and Arf4 mutant (bottom) embryos. Scale bar is 5 microns and applies to both images. E. Megalin staining (green) is normally concentrated at the apical surface of the visceral endoderm of E8.5 embryos. Mutants have significantly reduced megalin staining. Golgi organization (giantin, red) does not appear to be affected. F. Co-IP experiments showing GFP tagged fibrocystin (left two columns) and megalin (right two columns) expressed in cell lysates (GFP blot, top panel) along with FLAG tagged Arf4 (lanes 1 & 3) or Arf5 (lanes 2 & 4). Following immunoprecipitation of Arf4 and Arf5 (FLAG blot, bottom panel), Arf4 brought down fibrocystin and megalin (GFP blot, middle panel, lane 1 and 3) while Arf5 did not precipitate either fibrocystin or megalin (GFP blot, lanes 2 & 4, middle panel).

As *Arf4* is most highly expressed within the visceral endoderm during development, we examined this tissue further by immunofluorescence and electron microscopy (Figure 6). To determine if these tissues were ciliated, we stained yolk sacs and sectioned embryos for cilia, and imaged by confocal microscopy. We did not detect cilia on the visceral endoderm at embryonic day 8.5 or 9.5. However cilia are present on the adjacent inner layer of mesoderm cells in both wild type and mutant embryos at these stages in development (Figure 6A, B).

The visceral endoderm serves as the major secretory and absorptive tissue of the developing embryo prior to placental formation [25]. As expected for a highly absorptive tissue, the visceral endoderm has a well-developed brush border on the apical surface and large apical vacuoles/lysosomes that facilitate uptake and breakdown of macronutrients from the maternal blood supply (Figure 6C). In *Arf4* mutants the apical/basolateral polarity appears intact and the brush border remains, but the microvilli are less organized and the apical vacuoles are missing, suggesting that visceral endoderm function may be compromised. In addition, bulbous misshapen microvilli are often observed along with small vesicles that are surrounded by a fuzzy coat, which is not seen on the microvilli (Figure 6C, middle row). The cell-cell contacts also appear to be compromised as more space is seen between the mutant cells by TEM; moreover microprojections that form the interdigitations between the lateral surfaces of adjacent cells can be observed by SEM on the apical surface of the mutants but not the controls (Figure 6C, bottom row). It is interesting to note that the amnion is not detectably altered in the Arf4 mutants (Figure 6D) indicating that not all mutant cells are disrupted by the lack of Arf4.

The visceral endoderm carries out its absorptive function by localizing megalin (Lrp2) and other scavenger receptors on its apical surface. These receptors bind to substrates such as vitamins, lipoproteins and signaling molecules (reviewed in [26]), which are then internalized and transcytosed or broken down in the lysosome. Trafficking defects within the visceral endoderm result in embryonic lethality and are often associated with mislocalization of megalin [27], [28]. At E8.5, megalin is normally concentrated along the apical surface of the visceral endoderm (Figure 6E). Arf4 mutants have significantly reduced megalin staining at the apical surface and some of the protein appears in the cytoplasm. This suggests megalin trafficking is disrupted in *Arf4* mutants and supports the ultrastructural studies of the visceral endoderm that indicate nutrient uptake is compromised within this cell layer.

Megalin is a type 1 membrane protein with a large extra cellular domain, single transmembrane span and a short cytoplasmic C-terminal tail similar to the structure of fibrocystin. Because of the similarity in structure and the observation that *Arf4* mutant embryos have reduced megalin on the apical surface of the visceral endoderm, we asked if Arf4 interacts with megalin. Co-immunoprecipitation indicates Arf4 interacts with the intracellular domain of megalin (Figure 6F). Similar to what we observed with fibrocystin, the highly similar protein Arf5 did not interact with megalin. These data suggest Arf4 is involved in not just trafficking of ciliary cargo but also a larger class of trans-membrane receptors – including megalin.

## Discussion

The primary cilium is a sensory organelle and its proper function relies on the correct complement of receptors localized specifically in the ciliary membrane. Little is known about how proteins are sorted to the ciliary compartment but understanding this process is critical as defects in the signaling functions of primary cilia underlie a diverse group of human pathologies known collectively as ciliopathies. These diseases range from developmental defects of the brain, heart and other organs to chronic ailments including retinal degeneration, obesity and polycystic kidney disease. To study ciliary protein sorting, we focused on analysis of the trafficking of the transmembrane protein fibrocystin to the primary cilium. Mutations in the fibrocystin gene (*PKHD1*) are responsible for autosomal recessive polycystic kidney disease, a disorder afflicting approximately 1 in 20,000 individuals and a cause of significant mortality during the first year of life [29], [30]. The ciliary targeting sequence of fibrocystin is an 18 amino acid sequence contained in the cytoplasmic tail [7]. We had previously shown that the CTS interacts with the small G protein Rab8. In this work we studied the interaction of the CTS with another small G protein, Arf4. Arf proteins group vesicular cargo and through interactions with coat proteins form transport vesicles [12]–[14]. The proposed sorting ability of Arf proteins make them attractive candidates as specificity factors and recent work suggests Arf4 is involved in targeting rhodopsin and polycystin-1 to the cilium [10], [11], [18]. We found that Arf4 was capable of interacting with the fibrocystin CTS in a co-immunoprecipitation assay. The interaction is specific as Arf4 is the only member of this highly conserved family that precipitated significant amounts of the CTS. The analysis of deletion and alanine scanning mutants within the cytoplasmic tail of fibrocystin showed that the Arf4 interaction site was localized within the CTS. The ability of Arf4 to bind to the mutated CTSs roughly correlated to the ability of the CTS to enter the cilium suggesting that the Arf4/CTS interaction was functionally important.

Using SNAP-tagging technology we found that Arf4 is not absolutely required for the delivery of the fibrocystin targeting construct to cilia as the steady state level of the protein was not affected by knockdown of Arf4. This is in contrast to the reported effect of knockdown on the trafficking of polycystin-1 where Arf4 knockdown significantly reduced ciliary levels of polycystin-1 [18]. However, in our hands, the delivery of newly synthesized fibrocystin fusion protein was slower in the knockdown cells indicating that Arf4 is needed for the efficient delivery to primary cilia. The delayed, but eventual delivery of the CTS to the cilium may be a result of residual Arf4 protein (∼10% of the mRNA remains in the knockdown cells) or it may indicate an alternative pathway to the cilium that does not utilize Arf4. Smoothened appears to enter the ciliary compartment by first traveling to the plasma membrane before moving into the cilium [21] and so it is possible that this route can also be utilized by fibrocystin.

Our work, and work from others, indicates that Arf4 may play a key role in sorting transmembrane receptors to the cilium. This suggests that Arf4 may be an important player in human diseases such as retinal degeneration and polycystic kidney disease. To better understand the function of Arf4 and its possible role in ciliopathies, we created an *Arf4* mutant mouse. If the primary function of Arf4 is specific to cilia, we would expect the mutant mice to exhibit phenotypes in common with established mutations that affect cilia. Mice with strong defects in ciliary assembly die at mid-gestation with severe left-right abnormalities while those with more mild ciliary defects survive longer and display phenotypes indicative of hedgehog signaling dysfunction. *Arf4* mutant mice die between embryonic days 9 and 10, which is similar to the time when mice with severe ciliary assembly defects die. However, ciliary assembly is normal in the embryonic node and the nodal cilia are functional as all embryos broke left-right symmetry properly and formed a D-looped heart. The embryonic lethality but lack of ciliary defects suggested Arf4 might have functions in addition to the ciliary targeting of transmembrane proteins. Expression analysis between embryonic day 7 and 10, around the time the *Arf4* mutant embryos were dying, indicated that the major site of *Arf4* expression was in the visceral endoderm. Examination of the visceral endoderm indicates this tissue is not ciliated at this time in development although cilia are present on the adjacent mesoderm. The visceral endoderm is the major secretory and absorptive tissue of the developing embryo prior to chorioallantoic placenta formation [24] and defects within this cell layer often result in embryonic lethality [27], [28]. *Arf4* mutant embryos have multiple defects within the visceral endoderm. Ultrastructural analysis of the visceral endoderm indicates *Arf4* mutant embryos have defects in cell-cell contacts and organization of the brush border, but most strikingly, they lack the large lysosomes normally present in healthy tissue. The absence of lysosomes could be a direct effect of the *Arf4* mutation, but is more likely an indirect consequence of a failure to absorb nutrients from the adjacent maternal blood supply. A failure to uptake nutrients is consistent with the observed growth restriction evident by embryonic day 7 and likely accounts for the lethality around day 9. The apical surface of the visceral endoderm is covered with microvilli that contain a number of scavenger receptors that bind ligands including vitamins and lipoproteins required by the developing embryo. We examined the distribution of one of these receptors, megalin within the visceral endoderm. Megalin is normally localized to the apical surface of the developing visceral endoderm however in *Arf4* mutants, megalin fails to localize to the apical surface. Megalin is a large single span transmembrane receptor with membrane topology similar to fibrocystin. Co-immunoprecipitation assays indicate that Arf4 can interact with megalin similar to what we observed between Arf4 and fibrocystin. This interaction and the observed defects in megalin trafficking in the *Arf4* mutant suggest that Arf4 is required to target megalin to the apical surface. While our work was in progress, another gene trap allele of *Arf4* was generated and used to study dendritic spine formation in the brain. Similar to our allele, homozygotes were lost prior to birth but no further analysis was done to characterize the homozygous phenotypes [31].

RNAi studies suggested that the Arf family of proteins was highly redundant and it was not predicted that the genetic loss of any one would have a strong phenotype [32], [33]. The observation that *Arf4* null mice die mid gestation indicates that this is not completely correct. To date, *Arf6* is the only other Arf family member that has been mutated in the mouse. Like *Arf4*, *Arf6* null mice die during development, although they survive longer than the *Arf4* mice. The major defect in *Arf6* null mice was in the liver, where the lack of Arf6 caused increased rates of apoptosis resulting in a significantly smaller liver with lethality around embryonic day 15. [34]. The fact that both *Arf4* and *Arf6* mice survive through early gestation suggests that neither of these genes are essential genes at the cellular level, but do play critical functions in particular cells at particular times in development. In the case of the *Arf4* mouse, the major defect was in the visceral endoderm, a tissue with a very high rate of internalization and trafficking of lipid and protein molecules. It is possible that this is the first point in development that requires this level of internalization and trafficking. The analysis of a floxed allele will be of interest to determine if Arf4 is required in adult cells with a high rate of flux such as the intestine or kidney proximal tubule, or even the rod and cone photoreceptor cells where a high flux is needed to maintain the outer segment.

The literature on Arf4 has mostly focused on proposed roles in trafficking proteins to the ciliary membrane compartment. However, our finding that the highest level of expression is in the visceral endoderm, which is not ciliated at the time of high expression, suggests that Arf4 has functions outside the targeting of ciliary cargo. This is consistent with recent studies showing a role for Arf4 in Golgi stress responses [35] and the finding that class II Arfs (Arf4, Arf5) play roles in the trafficking of dense core vesicles [36], [37] and secretion of Dengue virus particles [33]. In the case of the dense core vesicle transport, Arf4 and Arf5 interacted with two calcium dependent activator proteins for secretion (CAPS1 and CAPS2). This interaction was required for the efficient trafficking of dense core vesicles as knockdown of either Arf4/5 or CAPS1/2 significantly reduced chromogranin secretion [36], [37]. In the case of Dengue virus production, Arf4 and Arf5 were required for the secretion of subviral particles and the Arfs were thought to act though an interaction with prM glycoprotein of the virus. Interestingly, the prM glycoprotein contains a VXPX motif in the C-terminus similar to the Arf4 binding site in rhodopsin [10]. However mutation of the VXPX motif did not disrupt interaction with Arf4 indicating that it is not the binding site [33]. This is consistent with our finding that Arf4 binds to the CTS of fibrocystin, which does not contain a VXPX motif and studies of nephrocystin-3, which contains a VXPX motif, but this motif is not necessary for ciliary targeting [38].

In conclusion, we have shown that Arf4 plays a role in the efficient transport of the fibrocystin CTS to the cilium, but it is not required for ciliary assembly and in the mouse has critical functions in non-ciliated cells. Thus, our work, and other published work, suggests that Arf4 function is not restricted to ciliary assembly but rather plays a broader role in cellular trafficking.

## Materials and Methods

### Ethics Statement

Mouse work was approved by the University of Massachusetts Medical School IACUC.

### Mouse Breeding

An *Arf4*-mutant ES cell line was obtained from the Sanger Center and used to generate *Arf4* ^Gt(AY0614)Wtsi^ mutant mice. The animals used in this study were a mix of 129 and C57Bl6 backgrounds. Embryonic ages were determined by timed mating with the day of the plug being embryonic day 0.5. Genotyping was carried out with the following primer pairs: Arf4-1 AGCAGCCTCATTGTCCTAGC+Arf4-2 CCTCCCCACAATTCAACAAT (product size = 189 bp in wildtype) and Geo-3 GATCGGCCATTGAACAAGAT+Geo-4 CAATAGCAGCCAGTCCCTTC (product size = 280 bp in mutant).

### Mammalian Cell Culture

IMCD3 (ATCC) were grown in 47.5% DMEM 47.5% F12, 5% fetal bovine serum, with penicillin and streptomycin at 37°C in 5% CO_2_. Cells were transfected by electroporation (Bio-Rad, Hercules CA). Stable cell lines were selected by supplementing the medium with 400 µg/ml of G418 (Sigma, St. Louis, MO). Clonal lines were selected by dilution cloning after drug selection.

### Electron Microscopy

For scanning electron microscopy (SEM), timed pregnant females were euthanized by approved IACUC protocols, embryos dissected in DMEM/F12 supplemented with 5% fetal bovine serum, fixed overnight in 2.5% glutaraldehyde in 0.1M sodium cacodylate. Fixed embryos were rinsed twice with 0.1M sodium cacodylate, osmicated in 1% osmium tetroxide, dehydrated in a graded ethanol series and critical point dried (Autosamdri-815, Series A, Tousimis Research Corp.). Dried embryos were sputter coated with iridium to a thickness of 3 nm (Cressington 208 HR Sputter Coater, Ted Pella, Redding, CA, USA) and examined in a scanning electron microscope (FEI Quanta 200 FEG SEM) [39]. For comparison of nodal cilia, embryos were developmentally matched by counting somite number.

For transmission electron microscopy (TEM), samples were fixed, osmicated and dehydrated as described above. Dehydrated samples were then infiltrated first with two changes of 100% propylene oxide and then with a 50%/50% propylene oxide/SPI-Pon 812 resin mixture. The following day, three changes of fresh 100% SPI-Pon 812 resin were done before the samples were polymerized at 68°C in plastic capsules. The samples were then reoriented and thin sections were placed on copper support grids and contrasted with Lead citrate and Uranyl acetate. Sections were examined using a Phillips CM10 TEM with 80 Kv accelerating voltage, and images were captured using a Gatan TEM CCD camera.

### Immunofluorescence Microscopy

Cells for immunofluorescence microscopy were grown, fixed, and stained as described [40]. For visceral endoderm immunofluorescence, E8.5 embryos were fixed for 15 minutes at room temperature with 4% paraformaldehyde in PBS rinsed twice in PBS, equilibrated in 30% sucrose overnight and embedded in Tissue Freezing Media (Triangle Biomedical Sciences). Cryosections (10 µm) were blocked for 1 hour in 1% bovine serum albumin, incubated with primary antibodies overnight at 4°C.

Primary antibodies included anti acetylated-tubulin (611B1, Sigma, St. Louis MO), anti-FLAG (Sigma), anti-MmIFT20, anti-MmIFT88 [41], MmIFT27 [42], anti-golgin97 (CDF4, Molecular Probes) anti-BIP (clone 40, BD Transduction Laboratories), anti-Rab11 (clone 47, BD Transduction Laboratories), anti-TfR (clone H68.4, Invitrogen), anti-giantin [43] and anti-megalin (P-20, Santa Cruz Biotechnology).

Widefield images were acquired by an Orca ER camera (Hamamatsu, Bridgewater, NJ) on a Zeiss Axiovert 200 M microscope equipped with a Zeiss 100× plan-Apochromat 1.4 NA objective. Images were captured by Openlab (Improvision, Waltham, MA) and adjusted for contrast in Adobe Photoshop. If comparisons are to be made between images, the photos were taken with identical conditions and manipulated equally. For the quantification of SNAP and CD8 in the cilia, the length, area, and average fluorescence intensity of the cilia was measured using the measurement tools of Openlab. To determine significance of differences, data from three independent experiments were subjected to an unpaired Student's T test. Confocal images were acquired by a Nikon TE-2000E2 inverted microscope equipped with a Solamere Technology modified Yokogawa CSU10 spinning disk confocal scan head. Z-stacks were acquired at 0.2 µm or 0.5 µm intervals and converted to single planes by maximum projection with MetaMorph software. Bright field images were acquired using a Zeiss Axioskop 2 Plus equipped with an Axiocam HRC color digital camera and Axiovision 4.0 acquisition software.

### SNAP Trafficking Assays

The construct (pJAF270) used for SNAP trafficking assays was constructed by fusing the extracellular domain of mouse CD8a [44] to the last 17 extracellular residues of mouse fibrocystin through the first 27 intracellular residues, the SNAP tag was cloned onto the c-terminal end of the CTS creating CD8-CTS-SNAP. Mouse kidney cells stably expressing CD8-CTS-SNAP were incubated with 0.04 µM cell permeable non-fluorescent BG-Block (New England Biolabs) for 30 minutes to block all SNAP epitopes. Following 3 washes with complete growth media cells were allowed to synthesize new CD8-CTS-SNAP for 1.5 hrs before the addition of HEPES pH 7.4 to 20 mM and cycloheximide to 150 µg/ml, then shifted to 19°C for two hrs to accumulate CD8-CTS-SNAP at the Golgi complex. Cells were returned to 37° and allowed to traffic CD8-CTS-SNAP for the indicated periods of time before being fixed and stained.

For siRNA knockdown, cells were transfected by RNAiMAXX (Invitrogen) with SMARTpool siRNA (Dharmacon) targeting Arf4 (L-060271) or a non-targeting control (D-001810) and assayed for knockdown 48 hours post transfection.

### Protein Analysis

FLAG-tagged Arf1-6 (pJAF215, pJAF213, pJAF214, pJAF216, pJAF210, pJAF211), were constructed by PCR amplifying the open reading frames and inserting them into p3XFLAG-CMV-14 (Sigma, St. Louis, MO). Point mutations in Arf4 (Arf4^T31N^ = pJAF221, Arf4^T48N^ = pJAF222, Arf4^Q71L^ = pJAF223) were generated by inverse PCR using the Quick Change II site directed mutagenesis kit (Stratagene) starting from pJAF216. Cells were transfected with FLAG-tagged Arf and GFP-tagged CTS deletion and alanine scanning mutants used in [7], CD8-PKHD1 (pJAF268), or CD8-Megalin (pJAF281) and 48 hours later, FLAG immunoprecipitation was carried out as described in [40].

### Beta-galactosidase Staining of Mouse Embryos

Embryos were fixed in 0.2% glutaraldehyde, 2% formalin, 5 mM EGTA and 2 mM MgCl_2_ in 0.1M phosphate buffer pH 7.3 for 10 minutes at room temperature then rinsed three times in wash buffer containing 0.1% sodium deoxycholate, 0.2% IGEPAL, 2 mM MgCl_2_ in 0.1M phosphate buffer for 30 minutes each wash. Fixed embryos were stained overnight at 37°C in 1 mg/ml X-gal, 5 mM potassium ferrocyanide, 5 mM potassium ferricyanide diluted in wash buffer.

### mRNA Analysis

RNA was isolated from individual E9.5 embryos or from IMCD3 cells using RNeasy kits (Qiagen), including on-column DNA digestion. First strand cDNA was synthesized from 100–500 ng of total RNA using a SuperScript II First-Strand Synthesis System (Invitrogen, Carlsbad, CA) and random hexameric primers. Quantitative real-time PCR primers were designed to produce amplicons between 100–150 nucleotides in length, using the online primer3 web PCR primer tool (http://fokker.wi.mit.edu/primer3/input.htm) and the IDT Primer Express software tool (http://www.idtdna.com/Scitools/Applications/Primerquest/). Primers were synthesized by Integrated DNA Technologies Inc (Coralville, IA) and are listed in Table 1. qRT-PCR analysis was performed using an ABI Prism 7500 sequence detection system (Applied Biosystems, Foster City, CA). Each reaction contained 5–12.5 ng first strand cDNA, 0.1 µM each specific forward and reverse primers and 1× Power SYBR Green (Applied Biosystems, Foster City, CA) in a 15 µl volume. Arf4 mRNA expression was normalized to GAPDH mRNA abundance and compared between mutant and control animals with an unpaired Student t-test.

**Table 1:** qPCR primers.

Primer Name	Sequence	Tm	Amplicon
MmArf4exon2FOR	GGATGCTGCTGGCAAGACGACA	62.2	138
MmArf4exon3REV	TGACCACCAACATCCCATACTGTGAAA	60	
MmArf4exon3FOR	TTCACAGTATGGGATGTTGGTGGTCA	59.9	133
MmArf4exon4REV	GCACAGCTGCTCCTTCCTGGATT	61.7	
MmArf4exon2FOR	GGATGCTGCTGGCAAGACGACA	62.2	245
MmArf4exon4REV	GCACAGCTGCTCCTTCCTGGATT	61.7	
MmArf4exon5FOR	CCAAACGCTATGGCCATCAGTGAGA	61.0	132
MmArf4exon6REV	TGACAGCCAATCCAGTCCCTCA	60.4	
MmGAPDHExon3FOR	GCAATGCATCCTGCACCACCA	61.1	138
MmGAPDHExon4rREV	TTCCAGAGGGGCCATCCACA	61.1	

## Supporting Information

Figure S1 Arf4 expression inhibits CTS trafficking. A. Mouse kidney cells stably expressing wild type (top row), dominant negative (T13N, T48N) and constitutively active (Q67L) Arf4-FLAG (red) stained with IFT20 (green) and DAPI (blue). Constitutively active (Q67L) localizes to the Golgi complex while wild type and dominant negative Arf4 are dispersed in the cytoplasm. B. Expression of dominant negative (T13N, T48N) Arf4 reduced the percent of ciliated cells. * p<0.05, ** p<0.01 as compared to IMCD cells not transfected with an Arf4-FLAG construct C. Dominant negative Arf4 (T13N, T48N) causes a reduction in ciliary length. * p<0.05, ** p<0.01 as compared to IMCD cells not transfected with an Arf4-FLAG construct D. Expression of any Arf4 constructs prevents ciliary trafficking of GFP-CTS. *** p<0.001 as compared to IMCD cells not transfected with an Arf4-FLAG construct. B–D, error bars are standard error of the mean. E. Co-immunoprecipitation experiments demonstrate that GFP-CTS interacts with FLAG-tagged Arf4 wild type and mutant proteins.
(TIF)

Figure S2 β-galactosidase stained mutant embryo dissected on embryonic day 7. Embryos were stained with X-gal, embedded in paraffin, sectioned, dewaxed and photographed. Planes are organized from proximal to the distal end of the conceptus. Panels A–C show extra-embryonic and panels D and E embryonic regions. Small arrows mark staining in the parietal endoderm and arrow heads mark the visceral endoderm.
(TIF)

Figure S3 β-galactosidase stained mutant embryo dissected on embryonic day 8. Embryos were stained with X-gal, embedded in paraffin, sectioned, dewaxed and photographed. Planes are organized from the proximal to distal regions of the conceptus. Panels A–C show extra-embryonic and panels D and E embryonic regions. Arrow heads mark the visceral endoderm. Small arrows mark staining in the allantois (plane B and C) or paraxial mesoderm (planes D and E). In plane D, the larger arrow marks the site where definitive endoderm is forming in the developing hindgut.
(TIF)
