# Supplementary figures and images for: Arf4 Is Required for Mammalian Development but Dispensable for Ciliary Assembly
Source: PLoS Genet. 2014 Feb 20;10(2):e1004170. doi: 10.1371/journal.pgen.1004170 (PMC3930517; doi:10.1371/journal.pgen.1004170)

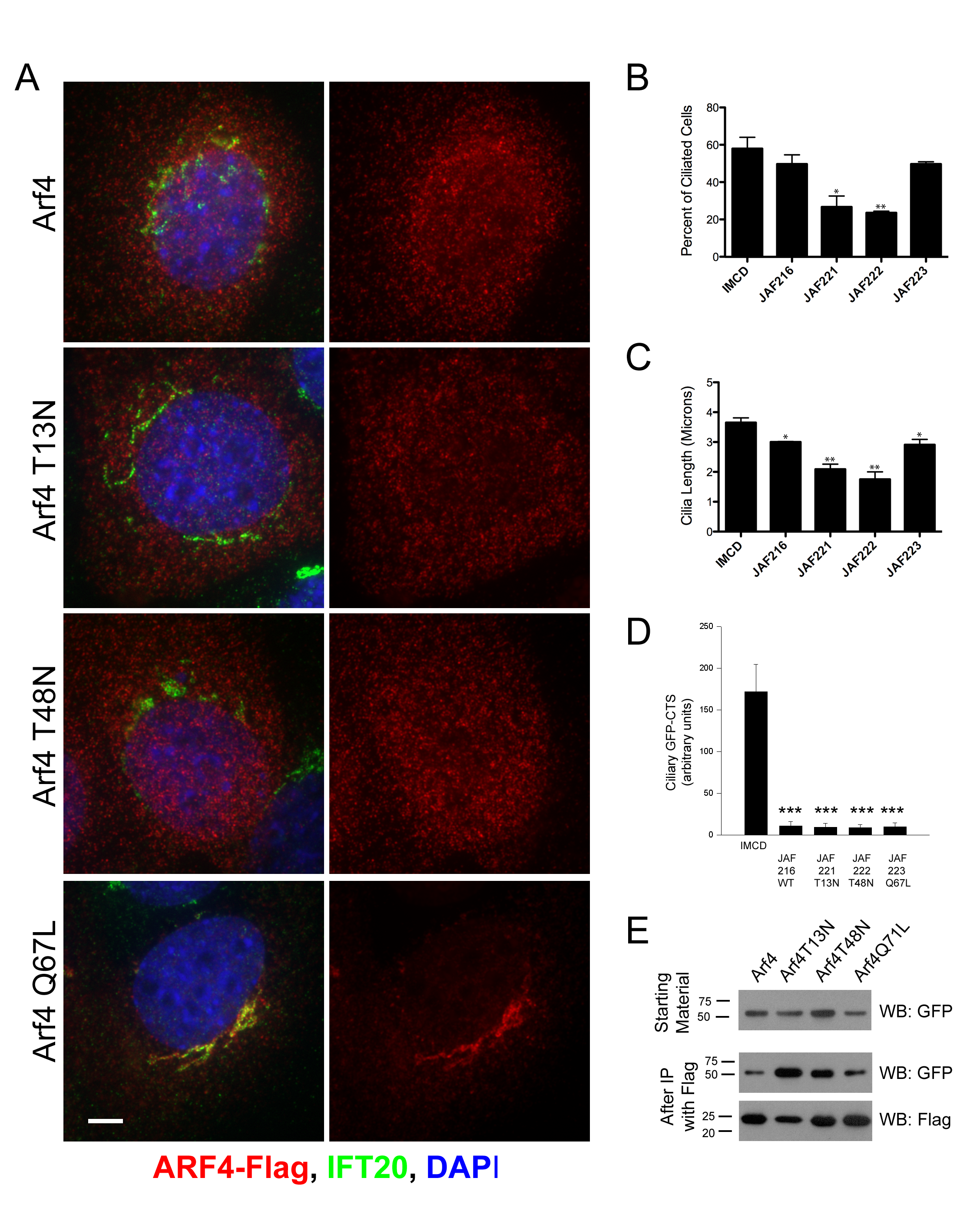

Supplement: Figure S1 — Arf4 expression inhibits CTS trafficking. A. Mouse kidney cells stably expressing wild type (top row), dominant negative (T13N, T48N) and constitutively active (Q67L) Arf4-FLAG (red) stained with IFT20 (green) and DAPI (blue). Constitutively active (Q67L) localizes to the Golgi complex while wild type and dominant negative Arf4 are dispersed in the cytoplasm. B. Expression of dominant negative (T13N, T48N) Arf4 reduced the percent of ciliated cells. * p<0.05, ** p<0.01 as compared to IMCD cells not transfected with an Arf4-FLAG construct C. Dominant negative Arf4 (T13N, T48N) causes a reduction in ciliary length. * p<0.05, ** p<0.01 as compared to IMCD cells not transfected with an Arf4-FLAG construct D. Expression of any Arf4 constructs prevents ciliary trafficking of GFP-CTS. *** p<0.001 as compared to IMCD cells not transfected with an Arf4-FLAG construct. B–D, error bars are standard error of the mean. E. Co-immunoprecipitation experiments demonstrate that GFP-CTS interacts with FLAG-tagged Arf4 wild type and mutant proteins. (TIF) [file pgen.1004170.s001.tif]

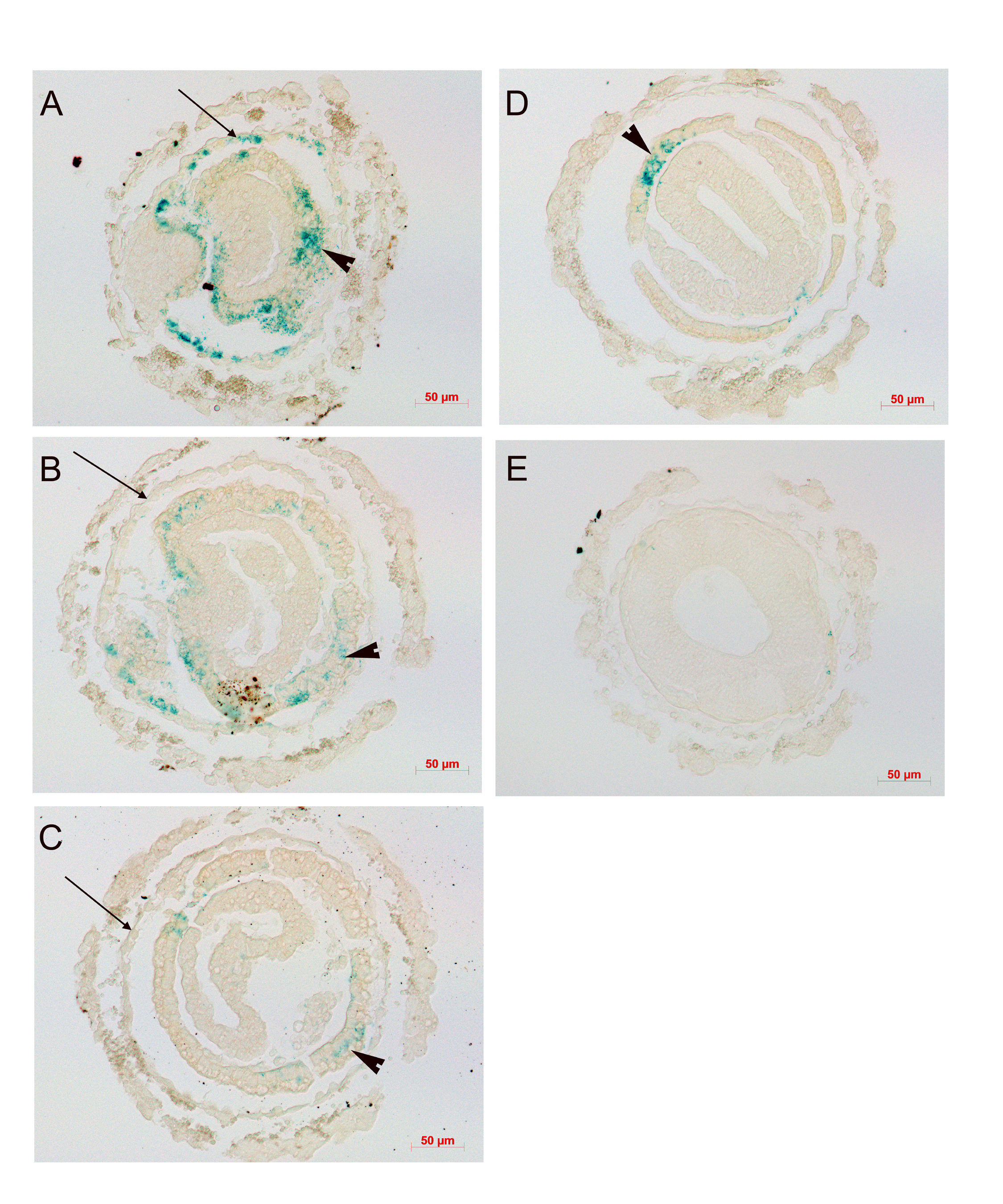

Supplement: Figure S2 — β-galactosidase stained mutant embryo dissected on embryonic day 7. Embryos were stained with X-gal, embedded in paraffin, sectioned, dewaxed and photographed. Planes are organized from proximal to the distal end of the conceptus. Panels A–C show extra-embryonic and panels D and E embryonic regions. Small arrows mark staining in the parietal endoderm and arrow heads mark the visceral endoderm. (TIF) [file pgen.1004170.s002.tif]

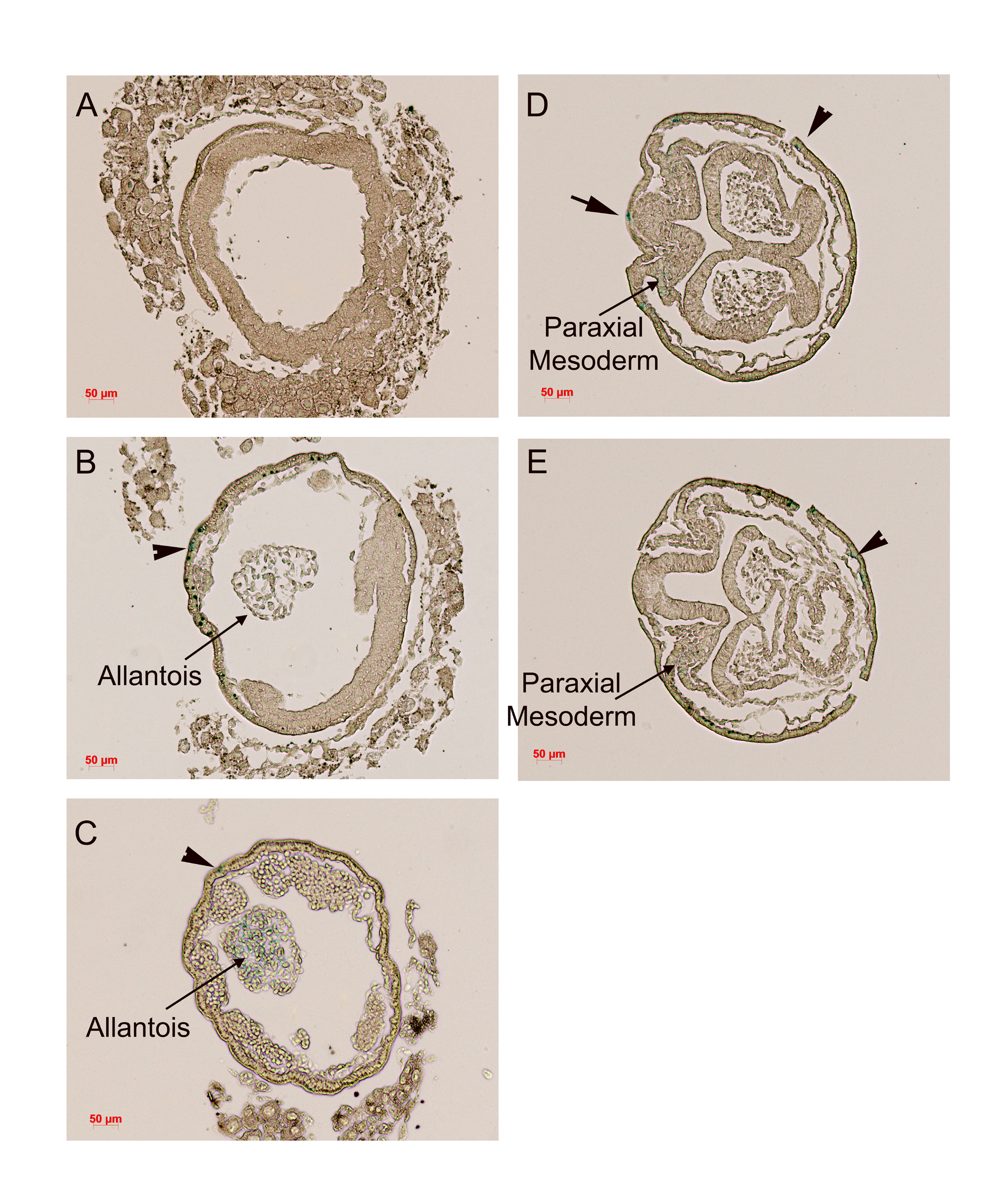

Supplement: Figure S3 — β-galactosidase stained mutant embryo dissected on embryonic day 8. Embryos were stained with X-gal, embedded in paraffin, sectioned, dewaxed and photographed. Planes are organized from the proximal to distal regions of the conceptus. Panels A–C show extra-embryonic and panels D and E embryonic regions. Arrow heads mark the visceral endoderm. Small arrows mark staining in the allantois (plane B and C) or paraxial mesoderm (planes D and E). In plane D, the larger arrow marks the site where definitive endoderm is forming in the developing hindgut. (TIF) [file pgen.1004170.s003.tif]
